# Mechanical Modulation, Physiological Roles, and Imaging Innovations of Intercellular Calcium Waves in Living Systems

**DOI:** 10.3390/cancers17111851

**Published:** 2025-05-31

**Authors:** Cole Mackey, Yuning Feng, Chenyu Liang, Angela Liang, He Tian, Om Prakash Narayan, Jiawei Dong, Yongchen Tai, Jingzhou Hu, Yu Mu, Quang Vo, Lizi Wu, Dietmar Siemann, Jing Pan, Xianrui Yang, Kejun Huang, Thomas George, Juan Guan, Xin Tang

**Affiliations:** 1 Department of Mechanical and Aerospace Engineering, Herbert Wertheim College of Engineering, University of Florida, Gainesville, FL 32611, USA; c.mackey1@ufl.edu (C.M.); feng.yuning@ufl.edu (Y.F.); liangc@ufl.edu (C.L.); jingpan@ufl.edu (J.P.); 2 Department of Chemistry, University of Florida, Gainesville, FL 32611, USA; 3 J. Crayton Pruitt Family Department of Biomedical Engineering, University of Florida, Gainesville, FL 32611, USA; a.liang@ufl.edu (A.L.); taiy@ufl.edu (Y.T.); 4 Independent Researcher, Watertown, MA 02472, USA; tianhe121@gmail.com; 5 Division of Chemical Biology & Medicinal Chemistry, College of Pharmacy, University of Texas at Austin, Austin, TX 78712, USA; om.arayan@utexas.edu (O.P.N.); jd55667@my.utexas.edu (J.D.); 6 Department of Computer and Information Science and Engineering, University of Florida, Gainesville, FL 32603, USA; jingzhouhu@ufl.edu (J.H.); kejun.huang@ufl.edu (K.H.); 7 Department of Molecular Genetics and Microbiology, University of Florida, Gainesville, FL 32603, USA; yumu@ufl.edu (Y.M.); lzwu@ufl.edu (L.W.); 8 Department of Biology, University of Florida, Gainesville, FL 32611, USA; quang.vo@ufl.edu; 9 University of Florida Health Cancer Center (UFHCC), Gainesville, FL 32610, USA; siemadw@ufl.edu (D.S.); thom.george@medicine.ufl.edu (T.G.); 10 College of Medicine, University of Florida, Gainesville, FL 32610, USA; 11 College of Dentistry, University of Florida, Gainesville, FL 32610, USA; xyang@dental.ufl.edu

**Keywords:** calcium waves, mechanobiology, cell–cell communications, functional imaging, mechanotransduction, AI/ML imaging analysis

## Abstract

Long-range intercellular communication is essential for multicellular biological systems to regulate multiscale cell–cell interactions and maintain life. Growing evidence suggests that intercellular calcium waves (ICWs) act as a class of long-range signals that influence a broad spectrum of cellular functions and behaviors. Importantly, mechanical signals, ranging from single-molecule-scale to tissue-scale in vivo, can initiate and modulate ICWs in addition to relatively well-appreciated biochemical and bioelectrical signals. Despite these recent conceptual and experimental advances, the full nature of underpinning mechanotransduction mechanisms by which cells convert mechanical signals into ICW dynamics remains poorly understood. This review provides a systematic analysis of quantitative ICW dynamics around three main stages: initiation, propagation, and regeneration/relay. We highlight the landscape of upstream molecules and organelles that sense and respond to mechanical stimuli, including mechanosensitive membrane proteins and cytoskeletal machinery. We clarify the roles of downstream molecular networks that mediate signal release, spread, and amplification, including adenosine triphosphate (ATP) release, purinergic receptor activation, and gap junction (GJ) communication. Furthermore, we discuss the broad pathophysiological implications of ICWs, covering pathophysiological processes such as cancer metastasis, tissue repair, and developmental patterning. Finally, we summarize recent advances in optical imaging and artificial intelligence (AI)/machine learning (ML) technologies that reveal the precise spatial-temporal-functional dynamics of ICWs and ATP waves. By synthesizing these insights, we offer a comprehensive framework of ICW mechanobiology and propose new directions for mechano-therapeutic strategies in disease diagnosis, cancer immunotherapies, and drug discovery.

## Simple Summary

Intercellular calcium waves (ICWs) are a class of molecular signals for long-range intercellular communication, influencing many key biological activities. Despite increasing evidence that mechanical signals across molecular to tissue scales initiate and modulate ICWs, the mechanisms by which cells transduce these forces into ICW-associated biochemical and genetic activities remain incompletely understood. This review discusses how mechanical stimuli interact with upstream molecules and organelles and how these signals propagate through downstream networks in living cells and tissues. By highlighting the roles of ICWs in various physiological and pathological contexts, with particular focus on cancer, this review proposes potential actionable mechano-therapeutic targets. Furthermore, this review summarizes recent advances in imaging and artificial intelligence technologies that have deepened the understanding of ICW dynamics. This review aims to offer a comprehensive framework of ICW mechanobiology and to highlight actionable mechano-therapeutic targets against ICW-related immune evasion and mechano-drug resistance in cancer.

## 1. Introduction

Mechanotransduction is the process by which cells sense and transduce mechanical stimuli into biochemical signaling and gene expression. It has a fundamental role in regulating cell functions and behaviors [1,2,3,4,5,6,7,8]. In particular, the mechanotransduction that occurs during multiscale cell–cell interactions influences diverse biological processes in animals [9,10,11,12,13,14], terrestrial plants [15,16,17,18], bacteria [19,20,21], and yeast [22,23,24]. Dysregulation of mechanotransduction is central to various pathologies characterized by aberrant cell–cell interactions, such as cancer metastasis and cardiac arrhythmia [12,14,25,26,27,28,29,30]. Therefore, a deeper understanding of how cells utilize mechanotransduction to maintain proper multiscale cell–cell interactions will provide key insights into the fundamentals of biology and bioengineering [30,31,32,33,34,35,36,37].

Calcium (Ca^2+^) is a crucial signaling messenger used by all eukaryotes [27,38,39,40,41]. Calcium signals impact nearly every aspect of cell biology across a wide range of spatial-temporal scales, from changes in protein conformation to functions of organelles, to the coordination of collective cell behaviors [42,43,44,45,46,47]. Disruptions in calcium signaling cause numerous human diseases, including but not limited to Alzheimer’s disease, heart failure, immunodeficiency, metabolic disorders, and cancer [48,49,50,51,52]. Intercellular calcium waves (ICWs) represent increases in cytoplasmic calcium ion concentration that occur in an initiating trigger cell among a cluster of cells and appear as propagating waves that radially transmit to the surrounding and distant cells [27,40,46,53]. Traditionally, electrically excitable cells such as neurons and myocytes are known to generate long-distance ICWs because they express diverse voltage-sensitive ion channels and proteins that enable transmitting fluctuations of membrane electrical voltage and triggering calcium influxes [54,55,56,57]. The excitable cells utilize ICWs as a fundamental mechanism for long-distance cell–cell interaction, with their electrophysiological mechanisms having been extensively investigated [58,59]. However, little is known about (1) whether and how electrically non-excitable cells such as epithelial cells produce spontaneous long-distance ICWs, and (2) whether and how non-electrical signals, especially mechanical forces, initiate and modulate ICWs. Because most cells in our bodies are electrically non-excitable, understanding ICW mechanobiology in non-excitable cells is warranted to discover new rules of life, promoting the creation of new therapeutic strategies which leverage mechanobiology principles.

In this review, we focus on reporting the quantitative interplay between mechanical signals and three main stages of ICW dynamics: initiation, propagation, and relay of long-distance ICWs. Several recent reviews have provided excellent summaries of the relationships between biochemical signals and ICWs [2,44,45,60,61]. However, a major knowledge gap is how cells sense and transduce the landscape of mechanical signals into ICWs and what the relationships are between the mechanical forces, cellular signaling, gene expression, and cell functions [62]. The goal of this review is to synergistically report (1) the recent advances in the understanding of mechanical influences in three main stages of ICW dynamics and the corresponding mechano-sensitive molecular effectors (Section 2); (2) the physiological roles of ICWs and ATP signals (Section 3); and (3) the recent technological advances in fluorescence-based calcium and ATP imaging and artificial intelligence (AI)/machine learning (ML)-enabled data analysis (Section 4). We conclude by establishing a new conceptual framework and proposing new paradigms on the future research directions of mechano-therapeutics to bridge the knowledge gap (Section 5).

## 2. Functional Interplay Between Mechanical Signals and Calcium Dynamics

To date, most long-distance ICWs observed in the electrically non-excitable cells were in cells cultured in stiff mechanical microenvironments (polystyrene (PS) petri dishes; elastic modulus = 3 GPa = 3 × 10^9^ Pa). Their initiation and propagation were triggered by the experimenter-applied mechanical, biochemical, or oncogenic stimuli [40,46,61,62,63,64,65,66]. As a result, these were not spontaneous. Interestingly, most reported spontaneous long-distance ICWs or increase of intracellular Ca^2+^ signals in non-excitable cells were observed in isolated tissues or during animal embryogenesis in vivo [67,68,69,70,71,72,73,74,75,76,77,78], where the mechanical microenvironment (10s kPa = 10 s × 10^3^ Pa) is 3–6 orders of magnitude softer than that in petri dishes in vitro. This phenotypic correlation inspires the hypothesis that mechanical signals in tissues may play a key regulatory role in the initiation, propagation, and function of ICWs. In Section 2, we analyze three main stages of ICW dynamics under the influence of mechanical signals and summarize their mechano-sensitive molecular effectors.

### 2.1. Mechanically Induced Initiation of ICWs

#### 2.1.1. Mechano-Regulated, Non-Spontaneous ICW Initiation

**Figure 1:**
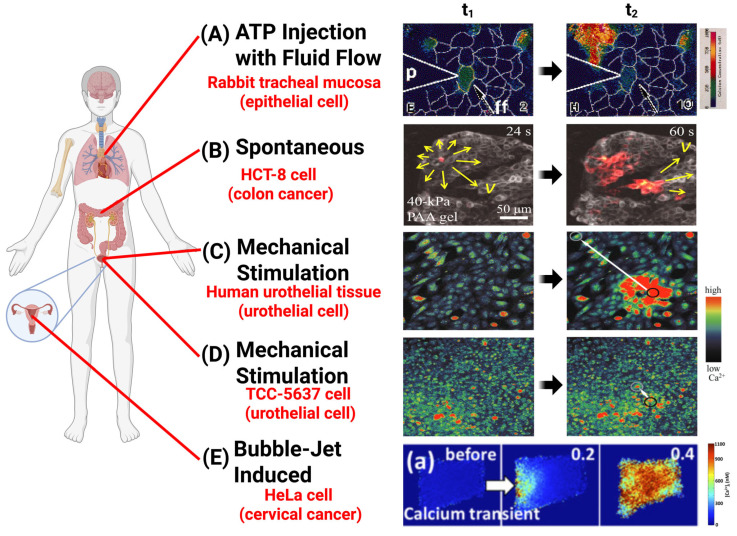
Intercellular calcium waves (ICWs) imaged in vitro in response to different forms of mechanical and biochemical stimulation. (**A**) [Ca^2+^] imaged in airway epithelial cell monolayer at t1 = 2 s and t2 = 10 s after local application of extracellular ATP in the presence of external fluid flow (ff, direction represented by white arrow) [93]. (**B**) Spontaneous ICW imaged in human HCT-8 colon cancer on 40-kPa polyacrylamide (PAA) gel at t1 = 24 s and t2 = 60 s. The yellow arrows indicate direction and velocity of wave propagation [53]. (**C**,**D**) [Ca^2+^] imaged in human urothelial cells and 5637 human transitional cell carcinoma (TCC) cells, respectively, at t1 = prior to single-cell micromanipulator stimulation and t2 = instance of maximal calcium wave distance propagation (white arrow) [85]. (**E**) Intracellular calcium transient progression imaged in singular HeLa cervical cancer cell in response to tandem bubble-induced jetting flow at t = 0, 0.2, and 0.4 s, respectively [89].

In research laboratories, external mechanical forces are widely applied in experiments to induce intracellular calcium dynamics and to reveal new signaling pathways (Figure 1). Utilizing different methods of mechanical application, these multiscale forces have been reported to activate calcium activities demonstrating a broad spectrum of spatial-temporal-functional dynamics (Table 1 and Table 2).

**Table 1:** Summary of available force application methods that activate calcium signals in distinct cell types and their physiological functions.

Category of Force Types	Methods of Application	Key Parameters	Molecular Mechanisms	Function
**Molecular-Scale Mechanical Stimulation**	Optical Laser Tweezers	Fibronectin (Fn)-coated bead (diameter = 10 μm), force = 300 pN [79]	Induce Ca^2+^ signals at the plasma membrane and ER via TRPM7 mechanosensitive channels.	ICWs in MSCs depend on TRPM7-mediated calcium signaling, which regulates differentiation.
	Light-Activated Molecular Machines	Forces = 10^−12^ to 10^−9^ N, laser = 400–405 nm (3.2 × 10^2^ to 9.0 × 10^2^ W/cm^2^), duration = 250 ms (in vitro), 1–2 s (Hydra) [80]	Induce ICWs via IP3-mediated signaling pathways.	MM-induced calcium wave generation can control biological behaviors coordinated in the networks of cells, such as contraction.
	Pipette Poking/Probing	Blunt-end glass micropipette, tip diameter = 50 μm [76]	ATP release via mechano-volume-sensitive Cl^−^ anion channels, activating receptors (P2X/P2Y) on wave-receiving cells.	Induced ICWs propagated at velocities of ~15 μm/s and distances of 200–300 μm, transmitting signals to adjacent cells.
	Scrape with Pipette Tip	Bent 200 μL micropipette tip [74]	ATP release stimulates calcium waves through purinergic receptor activation.	Induced ICWs regulated intercellular communication.
	Glass Micropipette with Micromanipulator	Micropipette tip coupled with 200 ng/mL EGF for 5 min [81]	EGF activates PLC-mediated calcium signaling.	-
		Tip diameter = 1 μm, movement = 2–5 μm, controlled by a piezoelectric device [82]	IP3 moves between gap junctions in epithelial respiratory tract cells.	-
		Tip diameter < 1 μm, touching less than 1 % of the cell membrane [83]	Induces a rapid Ca^2+^ spike and ICWs through gap junctions.	ICWs facilitate intercellular communication, regulate responses to mechanical and metabolic stress, and maintain metabolic homeostasis.
		Tip diameter of 1 μm, moved downward by 10 μm over 0.5 s coupled with 3 M KCl, delivered at 150 hPa for 1 s [84]	Ultrafast wave of calcium, traveling at approximately 15 mm/s.	ICWs synchronize contraction, regulate blood flow, and coordinate rapid vasomotor responses in SMCs.
	Microinjector Capillary	Micrometer precision [85]	ICWs are inhibited by GJ-blocking heptanol, indicating gap junction dependence.	ICWs maintain intercellular communication and coordinated cellular responses in urothelial cells.
	Force Probe or 30-Gauge Syringe Needle	Force range = ~2–300 μN, stimulation duration = 20–2000 ms [86]	Not specified.	Induced ICWs regulate endothelial communication, which is critical for immune modulation and tissue healing.
	Glass Microelectrode	Tip diameter = 1 μm [87]	Not specified.	-
**Subcellular/Cellular-Scales Mechanical Stimulation**	Focused Ultrasound (FUS)	Amplitude = 46 MHz (12 Vp–p), pulse repetition frequency = 1 kHz, duty cycle = 5% [88]	PANX1 mechanosensitive channels mediate calcium wave (propagation distance > 1 mm) initiation.	FUS-induced ICWs in PC-3 cells promote ATP release and cytokine/chemokine secretion via PANX1.
	Bubble-Jetting Methods	RGD-coated beads (6 μm), γ = Sd/Rmax = 1.2 to 2.4 (Sd = 30–60 μm) [89]	Intracellular calcium waves elicited by tandem bubble-induced jetting flow.	The bubble-induced rapid Ca^2+^ influx showed loss of F-actin stress fibers, cell shrinkage, and apoptosis.
	Parallel-Plate Flow Chamber	Shear stress from 100 to 400 μN/cm^2^ for 3 s [90]	Raising shear stress induced localized ATP release from caveolin-1-rich membrane domains, which activated purinergic receptors and initiated intracellular Ca^2+^ waves.	The shear stress triggered Ca^2+^ wave in HPAECs; contributed to cell shear-sensing.
**Tissue-Scale Mechanical Stimulation**	Mechanical Stretching	Stretching speed at 100 μm/s and distance at 200 μm (17.5% elongation) [91]	Stimulate Piezo1-dependent calcium influx and ATP release.	
	Applied Mechanical Loading	Diaphragm backpressure = 15 kPa, duration = 300 s [92]	Induces ICWs through physical deformation and ATP release.	ICWs regulate organ growth through calcium spikes, transients, and waves.

**Table 2:** Summary of diverse mechanical stimuli and corresponding molecular transducers that regulate different propagation stages of ICWs.

Mechanical Force Type	Molecular Transducers	Mechanotransduction Pathway
**Shear Stress**	Caveolin-1, P2X/P2Y purinergic receptors; Integrins	Shear stress induces ATP release from caveolin-1-enriched membrane domains, which activates P2X/P2Y purinergic receptors and initiates ICWs [90]. Similarly, laser-induced tandem bubble-jetting flow activates integrins, the mechanosensitive ion channel TRPM7, leading to calcium influx and subsequent ER-mediated calcium-induced calcium release [89].
**Tension and Stretch**	Integrins, Piezo1, Gq-PLC-IP3R pathway	Mechanical stretching or increased ECM stiffness is sensed by integrins and Gq-PLC-IP3R pathway-mediated calcium release [53,94,95,96]; Piezo1 mediates direct calcium influx under stretch-induced membrane tension [97,98,99,100].
**Point Stress and Compression**	TRPM7, Cl^−^ channels, IP_3_R, P2X/P2Y receptors, Connexin-based Gap Junctions	Local mechanical indentation activates volume-sensitive Cl^−^ channels and TRPM7, resulting in ATP release and P2X/P2Y purinergic receptor activation [74,76,79]; IP_3_ is generated and triggers IP_3_R-mediated calcium release [81,82,86], which propagates through GJ [83,84,85,87].
**Membrane Tension**	Piezo1 (Force-from-Lipids); PANX1 (ER), IP_3_R (Ultrasound-induced ER deformation)	In cytoskeleton-deficient conditions, membrane tension activates Piezo1 through a force-from-lipids mechanism, leading to calcium influx [101]; FUS induces ER membrane deformation, activating PANX1 channels and IP_3_Rs to release calcium [88,102].
**Stress Relaxation**	IP_3_R, Inx2 (Gap Junctions in *Drosophila*)	Mechanical stress release leads to the generation of IP_3_, which activates IP_3_Rs and promotes calcium release from the ER and propagation through GJs (Inx2) [92,103].
**Nanoscale Molecular Force**	IP_3_R	Light-activated molecular machines deliver nanoscale rotational forces, stimulating Gq-PLC-IP_3_ pathway and leading to calcium release and ICW generation [80].

**Figure 2:**
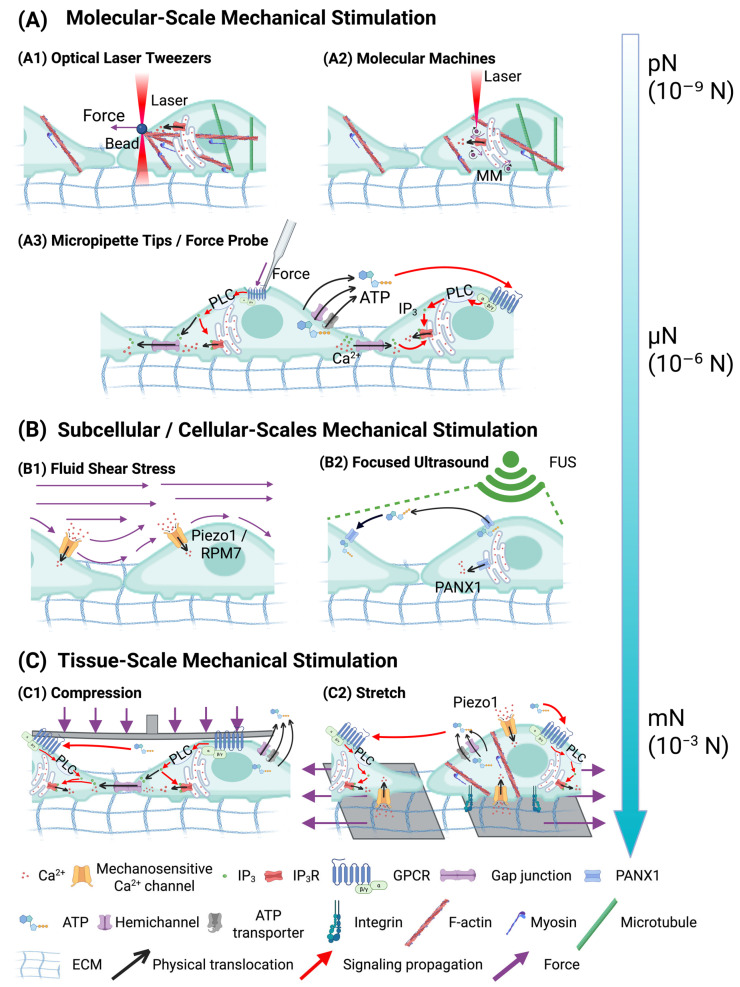
Mechanical stimulation methods across multiple scales regulate calcium and ATP signaling. The force range spans from piconewtons (pN) at the molecular level to millinewtons (mN) at the tissue level. (**A**) Molecular-scale mechanical stimulation. (**A1**) Optical laser tweezers apply force via beads [79]. (**A2**) Light-activated molecular machines (MM) stimulate intracellular calcium signaling [80]. (**A3**) Micropipette tips or force probes mechanically trigger mechano-volume-sensitive Cl- anion channels and PLC-IP3R-mediated calcium release and ATP propagation [74,76,81,85]. (**B**) Subcellular/cellular-scale mechanical stimulation. (**B1**) Fluid shear stress activating mechanosensitive channels like Piezo1 and TRPM7 [89]. (**B2**) Focused ultrasound (FUS) directly stimulates PANX1 hemichannels, facilitating intercellular calcium release [88]. (**C**) Tissue-scale mechanical stimulation includes (**C1**) compression and (**C2**) stretch, both enhancing calcium signaling via mechanosensitive channels and PLC-IP3R activation [91,92].

At the molecular scale, mechanical stimuli that are applied by precise and minimally invasive tools primarily target subcellular structures (Figure 2A). For example, a light-activated fast-rotating molecular machine (MM) can be delivered into cytoplasm to exert unidirectional forces in the range of 10^−12^ N (=1 pN) to 10^−9^ N (=1 nN) to stimulate ICWs through the pathway of inositol trisphosphate (IP3)-mediated signaling. This MM technology uses laser stimulation at 3.2 × 10^2^ W/cm^2^ (in vitro; 400 nm), 5.1 × 10^2^ W/cm^2^ (cardiomyocytes; 400 nm), and 9.0 × 10^2^ W/cm^2^ (Hydra; 405 nm) for 250 ms in vitro and 1–2 s for Hydra, depending on specific research goals [80]. Similarly, optical laser tweezers can apply a mechanical force of 300 pN to the fibronectin (Fn)-coated bead (10-μm diameter), which is attached to the plasma membrane. This force can induce Ca^2+^ signals at the plasma membrane and the endoplasmic reticulum (ER) in human mesenchymal stem cells (MSCs) [79].

Using a highly precise micropipette or force probe, contact-based mechanical stimulation can be achieved at a molecular scale, offering spatial precision in the micrometer and nanonewton range. Using a blunt-end glass micropipette with a tip diameter of 50 μm, mechanical poking or probing on local regions of cellular membrane in human DU-145 prostate cancer cells can induce intercellular propagation of ICWs, with wave velocities reaching approximately 15 μm/s and propagation distances achieving 200–300 μm. Some studies suggest that the propagation of Ca^2+^ waves occurs through the mechano-regulated secretion of ATP molecules from wave-initiating cells and the activation of purinergic receptors on wave-receiving distant cells. Mechanistically, the mechano-volume-sensitive Cl^−^ anion channels on cells are activated by cell swelling or local changes in cellular mechanical strain and trigger the release of ATP molecules [104]. Similarly, linear mechanically scraping epithelial cancer cells using a bent 200-μL micropipette tip stimulates extracellular ATP release and initial calcium waves through the activation of purinergic receptors on distant cells [74]. Additionally, using a micromanipulator-controlled glass micropipette to perform mechanical stimulation, coupled with chemical epidermal growth factors (EGF) via EGF receptors (EGFR) at a concentration of 200 ng/mL for 5 min, intracellular calcium transients and intercellular calcium waves are activated in MDA-MB-468 cells [81]. A piezoelectric device driving a fine glass microprobe with a tip diameter of 1 μm and precise movements of 2–5 μm induces intracellular Ca^2+^ levels in epithelial cells via the movement of IP3 through GJs between epithelial respiratory tract cells [82]. The combination of microinjector capillary and pharmacological GJ (gap junctions)-blocking heptanol reveals the GJ-dependent propagation mechanism of ICWs [85]. The force probe or 30-gauge syringe needle can apply forces ranging from ~2–300 μN with a stimulation duration of 20–2000 ms on cells and induce calcium waves [85]. Glass microelectrodes with a tip of 1 μm diameter have been employed to investigate calcium communication induced by mechanical force [87]. Briefly touching less than 1 % of the cell membrane with a glass micropipette (tip diameter  <  1 μm) induces a rapid intracellular Ca^2+^ spike, which spreads to neighboring cells. This technique, applied to bovine corneal endothelial cells (BCEC), enables the study of intercellular communication and GJ function [83]. Similarly, ICWs in vascular smooth muscle cells (SMCs) were triggered by either mechanical stimulation applied locally using a micropipette (tip diameter of 1 μm, moved downward by 10 μm over 0.5 s) or chemical stimulation using locally applied KCl (3 M KCl, delivered under controlled pressure of 15 kPa for 1 s). These stimuli depolarize the membrane, allow the propagation of electrical signals into neighboring cells, and consequentially activate voltage-gated calcium channels to facilitate calcium influx. The resulting ultrafast calcium waves, traveling at approximately 15 mm/s, were inhibited when GJs between cells or voltage-operated calcium channels were blocked [84].

Together, these methods provide precise, physiologically relevant, and minimally invasive ways to dissect the fundamental roles of cellular mechanical forces in initiating non-spontaneous intracellular calcium signaling and propagating ICWs, demonstrating how mechanical forces are translated into calcium signaling at the smallest biological scales.

At the subcellular and cellular scales, a variety of methods demonstrate how forces can induce ICWs from organelles and influence cellular behaviors (Figure 2B). Hydrodynamic forces generated by bubble-jetting methods, which use RGD-coated beads of 6-μm and combine with single-bubble treatment (γ = Sd/Rmax = 1.2 to 2.4 (Sd = 30 μm to 60 μm)), can elicit the propagation of a calcium wave in HeLa cells by activating integrins, which triggers the mechanosensitive ion channel TRPM7, leading to calcium influx and subsequent ER-mediated calcium-induced calcium release. [89]. Similarly, in human pulmonary artery endothelial cells (HPAECs) that are exposed to shear stress in a parallel-plate flow chamber (100 to 400 μN/cm^2^ for 3 s), highly localized ATP release occurs at caveolin-1-rich plasma membrane domains (caveolae), which activates nearby P2X/P2Y purinergic receptors and triggers intracellular Ca^2+^ increases that initiate Ca^2+^ waves propagating across the cell monolayer [90]. On the subcellular level, a focused ultrasound (FUS) that has an amplitude of 46 MHz (12 Vp–p), pulse repetition of 1 kHz frequency, and duty of 5% cycle, stimulates ICWs in PC-3 prostate cancer cells via mechanosensitive pannexin-1 (PANX1) channels in the ER without cytoskeletal dependence [88]. Importantly, the FUS-activated calcium elevation has been reported for invasive prostate (PC-3 and DU-145) and bladder (T24/83) cancer cell lines, but not for non-invasive cell lines (BPH-1, PNT1A, and RT112/84). The ICWs propagate from the cells at the focus of the transducer to other cells over spatial distances greater than 1 mm [102]. These subcellular- and cellular-scale mechanical stimulations elucidate the roles of mechanical signals in activating organelle-based calcium waves and intercellular communications through distinct mechanosensitive pathways.

At the tissue and organ scales, macroscopic mechanical forces, including compression, stretching, shear stress, and membrane tension, can be transduced into intracellular and intercellular calcium signals (Figure 2C). The mechanosensitive Piezo1 channel has a critical role during this transduction in multiple cancer models, including gastric, breast, prostate, glioma, and osteosarcoma cancers [97,105]. Mechanical stretching of bladder urothelium cells with stretching speed at 100 μm/s and stretching displacement at 200 μm (actual cell extension rate, 17.5%) stimulates calcium influx and ATP release, which is dependent on Piezo1 activation [98]. Similarly, mechanical loading applied on discs of *Drosophila* wings at a diaphragm backpressure of 15 kPa for 300 s, such as the release of loads from external sources, has been shown to stimulate ICWs [92]. Together, tissue-scale mechanical forces, including stretching, compression, and shear stress, provide insights into the critical roles of physical deformation in triggering ATP release and calcium influx across heterogeneous cell populations, offering a framework for understanding system-wide mechanotransduction in both physiological and pathological processes.

Depending on the cell types and experimental settings, mechanical stimuli have dual roles in initiating ICWs, either as the primary driver or through the coordination with biochemical pathways. On the one hand, light-activated molecular machines, optical tweezers, and FUS can dominantly trigger ICWs via direct mechanotransduction pathways involving IP3 signaling, mechanosensitive ion channels (e.g., TRPM7, PANX1), or intracellular calcium mobilization. On the other hand, pipette scraping, shear stress, and membrane stretch as mechanical cues that rely on ATP release or EGF receptor activation subsequently propagate ICWs through purinergic signaling, receptor-mediated pathways, or GJ communication.

Comparison of current experimental models of mechanically induced ICWs reveals that most studies have been conducted in 2D monolayer cell models. This is mainly due to the advantages of 2D models, including ease of applying mechanical interventions, compatibility with optical imaging, and reproducibility. However, 2D models lack complex 3D architecture and mechanical microenvironments in physiological tissues.

In contrast, studies of mechanically induced ICWs in 3D in vitro, ex vivo, or in vivo models have been reported, but are very limited to date. For example, in an ex vivo *Drosophila* wing disc model cultured with 15% fly extract, the release of 15 kPa compressive mechanical stress via the regulated environment for microorgans chip (REM-Chip) device triggered IP_3_- and GJ-mediated ICWs [92]. Most studies using 3D models and in vivo models have focused on spontaneous ICWs or calcium waves induced by biochemical stimuli (e.g., KCl injection, caged-ATP release). This is mainly due to the more complex structures of 3D models, making it difficult to achieve both 3D precise mechanical control and high-resolution imaging simultaneously. These limitations highlight a critical technological gap and underscore the current need for new 3D model-based mechanobiological tools to deepen our understanding of physiologically relevant ICWs.

#### 2.1.2. Spontaneous Initiation Mechanisms of Multiscale ICWs

Spontaneous ICWs are a well-known phenomenon in electrically excitable cells including neurons [72,106], astrocytes [77,107,108,109], glial cells [46,87,110,111,112,113], and muscle cells [46,80,84,114,115,116]. In neurons, spontaneous ICWs have been linked to various developmental phenomena including regulation of neurite extension [117], guidance of neocortical growth [113], and neuronal differentiation [118]. Spontaneous ICWs have been linked to cochlear development in pre-hearing mammalian cochlea [119]. In extraocular muscles, spontaneous slow ICWs (max duration of 2–12 s, velocity of 25–50 μm/s) have been linked to localized myofiber contractions [115]. In smooth muscle cells, a mechanical signaling system that utilizes ECM stiffness to modulate calcium wave frequency and agonist sensitivity was identified, likely contributing to diseases like asthma and hypertension, independent of GJs or diffusing signals [114].

Spontaneous ICWs have infrequently been observed in cancer cell lines as well. HCT-8 colon cancer and DU145 prostate cancer were recently shown to generate spontaneous calcium transients and ICWs without active mechanical probing. In vitro cultures and ex vivo HCT-8 xenograft tumor slices revealed that both calcium transient duration and ICW propagation distance are influenced by substrate stiffness, with distinctly low and high substrate stiffnesses (250 Pa and 3 GPa) provoking decreased transient duration and propagation distance compared to intermediate stiffnesses (10, 20, and 40 kPa) [53]. These substrate-stiffness-dependent spontaneous ICWs were shown to modulate tumor growth in mouse models. Considering that mechanical stiffness of solid tumors is higher than that of healthy tissues, this finding potentially suggests a promoting role of tumor stiffness and ICWs on cancer progression.

Spontaneous ICWs also play a role in *Drosophila* organogenesis [92], tissue repair [120], smooth muscle differentiation [121], and actomyosin regulation [122]. In *Drosophila* imaginal wing disc development, spontaneous ICWs are regulated by biochemical signaling and release of mechanical stress [92]. Spontaneous oscillatory ICWs have been observed in *Drosophila* imaginal discs, where wave properties including initiation and oscillation frequency were found to be influenced by factors such as rearing temperature, cellular organization, and high regional Wingless (Wnt) signaling [122].

Finally, while intracellular Ca^2+^ oscillations lack the spreading characteristic of ICWs, these oscillations play a regulatory role in cell processes such as cell division, migration, fertilization, and apoptosis, which may shed light on the potential physiological impact of ICWs [42,45]. In human mesenchymal stem cells (HMSCs), spontaneous Ca^2+^ oscillations are shown to be regulated by extracellular substrate rigidity via the RhoA/ROCK signaling pathway, whereupon they influence cell differentiation outcomes [123]. In esophageal squamous cell carcinoma (ESCC), Orai1-mediated hyperactive intracellular Ca^2+^ oscillations have been shown to promote tumorigenic behavior such as cell proliferation, migration, and invasion in both in vitro cultures and in vivo xenografted mice [124].

### 2.2. Molecular Effectors Underpinning the Mechano-Regulated Initiation of ICWs

#### 2.2.1. Roles of Interacting Cytoskeleton and Mechanosensitive Ion Channels

Upon mechanical stimulation of a cell, the mechanical signal propagates through various force-sensing molecules and organelles. These effectors can be broadly categorized into three functional classes: (1) Mechanosensitive ion channels, such as transient receptor potential melastatin 7 (TRPM7) [79], Piezo-1 [97,98,99,100], pannexin-1 (PANX1) [88], connexin channels [125], and cytoskeletal components, which activate cascades of signal transduction to induce Ca^2+^ release from the ER and subsequent cytoplasmic Ca^2+^ spikes in adjacent cells, thus initiating an ICW [46,94]. (2) Intracellular calcium release channels, primarily the inositol 1,4,5-trisphosphate receptor (IP_3_R) [53,102,117,122], which mediate calcium release from the endoplasmic reticulum (ER) in response to IP_3_ binding. In some cases, calcium signaling can be tuned by mechanical substrate stiffness [53,123]. Importantly, increasing studies suggest that the participation of the cytoskeleton into these mechanotransduction processes can be case dependent [79,88].

Beyond ion channels, mechanotransduction also involves integrins and actin cytoskeleton. Acting as transmembrane receptors, integrins anchor cells to the extracellular matrix and serve as key mechanosensors. When mechanical forces like tension are applied to the ECM or cells sense high environmental stiffness, integrins cluster and activate intracellular signaling pathways [95]. These signals are transmitted to the cytoskeleton, including actin filaments, intermediate filaments, and microtubules, which reorganize and enable the adaptation of cellular shape, stiffness, and motility [96,126]. This dynamic feedback loop enables cells to sense mechanical cues and convert them into biochemical signals to regulate processes such as migration, proliferation, differentiation, ion channel gating, gene expression, and stem cell fate [127]. Some studies report that the cytoskeleton plays a critical role in the mechano-transduction process contributing to ICW initiation. In human mesenchymal stem cells (MSCs), the cytoskeletal network of contractile actomyosin machinery and microtubules were demonstrated to transmit mechanical force to the ER, potentially triggering Ca^2+^ release from the ER through activation of inositol 1,4,5-trisphosphate receptors (IP_3_Rs) or other transient receptor potential (TRP) family ion channels. Additionally, the mechanosensitive channel TRPM7, which transports Ca^2+^, Mg^2+^, and Zn^2+^ ions, was found to play a critical role. TRPM7 facilitates force transmission to the ER, exerts downstream effects on IP3R, or can be directly coupled to IP3R via adaptor proteins, including ankyrins [79]. In *Drosophila* imaginal discs, the relaxation of mechanical stress, rather than the stress itself, was found to initiate ICWs in a process dependent on IP3-mediated Ca^2+^ release from ER and propagation through GJs (Inx2) [92,103]. The temporal and spatial characteristics of the ICW response to mechanical loading and release were primarily dictated by baseline spontaneous ICW activity, with higher baseline levels correlating with prolonged burst duration (~275 s vs. ~125 s) and greater area fraction (~0.75 vs. ~0.25) [92].

On the other hand, some ICWs’ initial path from mechanical stimulation may bypass the cytoskeleton entirely. Mechanosensitive PANX1 channels localized in the ER mediate calcium release from internal stores in response to FUS, potentially due to membrane deformation induced by FUS acoustic pressure waves leading to PANX1 activation. This signaling pathway is independent of the cytoskeletal integrity in invasive PC-3 prostate cancer and HEK 293T human embryonic kidney cells and critically relies on both PANX1 and IP3R for ICW initiation and propagation [88]. However, how mechanical forces interact with PANX1 and IP3R on the ER remains to be determined. Similarly, in DU-145 prostate cancer cells, volume-sensitive anion channels in the plasma membrane mediate ATP secretion in response to pipette-based poking stimulation. During this process, the release of Ca^2+^ signals from the ER and the propagation of ICWs occur independently of the cytoskeleton, because the disruption of microfilaments and microtubules by cytochalasin B and nocodazole has shown no effect on propagation of calcium wave [76]. Additionally, Piezo1, a mechanosensitive ion channel [128,129], was activated using Yoda1 as a chemical substitute for mechanical stimulation in colon cancer stem-like cells (CCSCs), HCT-116, and HCT-8 cells. Activation of Piezo1 induced Ca^2+^ influx and subsequently activated NFAT1, which is a critical regulator for maintaining the stemness of CCSCs [99]. In cytoskeleton-deficient HEK293 cells’ membrane blebs, mechanical forces can activate the Piezo1 pathway via a “Force-from-Lipids” mechanism, inducing calcium ion influx [101].

Overall, these studies suggest that diverse mechanotransduction mechanisms can regulate ICW initiation and propagation, with or without cytoskeletal involvement. While the exact mechanisms by which mechanical stimulation is transduced into downstream ICW propagation remain unclear in many cell lines, exploring the mechanistic diversity and cell-type specificity of these pathways will promote a deeper understanding of how mechanical signals regulate calcium dynamics. Additionally, targeting specific mechanosensitive ion channels, such as TRPM7, Piezo1, or PANX1, may provide new therapeutic strategies for diseases associated with calcium signaling, such as cancer, heart diseases, and inflammation [130,131,132,133,134,135,136].

#### 2.2.2. Roles of IP3 and IP3R in Intracellular Calcium Release

Following the receiving of mechanical signals, IP3 activated by the Gq-PLC-IP3R pathway is frequently recognized as a direct effector for the initiation of intracellular calcium release. IP3 binds to the IP3 receptor (IP3R) on the ER, triggering the release of intracellular Ca^2+^ ions into the cytosol [53,92,94,122]. At low cytoplasmic concentrations, these initial Ca^2+^ ions can stimulate increased release of Ca^2+^ from the ER through biphasic modulation of IP3R channel gating, leading to Ca^2+^-induced Ca^2+^ release (CICR) [86]. Calcium diffusing from one open channel can trigger adjacent channels to open, amplifying the release in a self-reinforcing loop, which is eventually countered by inhibitory feedback at high Ca^2+^ concentrations [137]. Since both IP3 production and IP3R opening are subject to positive and negative feedback regulations by Ca^2+^ and IP3 in a concentration-dependent manner, with Ca^2+^ exerting both stimulatory and inhibitory effects, intracellular Ca^2+^ oscillations emerge from complex signal amplification and refractory dynamics [64,122]. Significantly, the remodeling of IP3R-mediated Ca^2+^ signaling is recognized as a central key mediator that controls the cellular processes in cancer progression [138].

Elevated substrate stiffness may increase plasma membrane tension, activating cytoskeletal proteins via integrins, actin filaments, and G-protein coupled receptors (GPCRs) including GPR68 and H1R. These activated GPCRs dissociate the G_q_ protein, whose alpha subunit activates Phospholipase-C (PLC) to cleave Phosphatidylinositol 4,5-bisphosphate (PIP2) into diacylglycerol (DAG) and IP3. This IP3 then activates IP3R to release ER Ca^2+^, raising [Ca^2+^]cyt. Alternatively, increased cytoskeletal tension on stiffer substrates could directly open IP3Rs through interaction with cytoskeletal proteins. Elevated [Ca^2+^]cyt subsequently activates cyclin D1/CDK4 and cyclin E/CDK2 signaling, facilitating the G1/S transition and promoting cancer cell proliferation and tumor growth in vivo [53,94]. However, how the mechanical signals mechanistically regulate the detailed spatial-temporal dynamics of these molecular networks to produce Ca^2+^ oscillation remains to be elucidated.

Upon release from intracellular stores such as the ER, calcium ions act as key initiators of intercellular communication by triggering a cascade of downstream signaling events. The initial release of calcium binds to calcium-binding proteins like calmodulin, which activates various kinases and phosphatases, including protein kinase C (PKC) and proto-oncogene tyrosine-protein kinase Src. Consequently, they modulate cellular processes such as gene expression, cytoskeletal remodeling, and cell proliferation [85,88]. Calcium influx is further facilitated by stretch-activated and store-operated channels, such as TRPM7, which are essential for mechanosensitive calcium entry into the cytoplasm [79]. This influx amplifies the cytosolic calcium concentration, initiating additional calcium release through CICR, thereby propagating the calcium signal across neighboring cells [85,86].

Ryanodine receptors (RyRs) are another channel protein responsible for releasing Ca^2+^ from the ER into the cytoplasm [139,140]. Unlike IP3Rs, however, RyRs are infrequently in ICWs of transient generations. RyR and TRP channels are essentially not expressed in *Drosophila* imaginal discs, suggesting that ICWs present are not propagated by any RyR-dependent mechanism [122]. Application of the RyR inhibitor dantrolene (Dan) had no effect on ICWs present in RasV12-transformed mammalian epithelial cells [27], nor did it affect Ca^2+^ transients in HCT-8 colon cancer cells [53]. However, while RyRs do not contribute to the IP3R-mediated Ca^2+^ release via CICR in single colonic smooth muscle cells, RyR blockers like ryanodine can still inhibit Ca^2+^ signaling if RyRs were already activated (e.g., by caffeine or depolarization), indirectly affecting IP3-mediated release by depleting the ER of Ca^2+^ [141].

### 2.3. Multiscale Release, Propagation, and Regeneration of ICWs

#### 2.3.1. Overview of Primary Mechanisms

**Figure 3:**
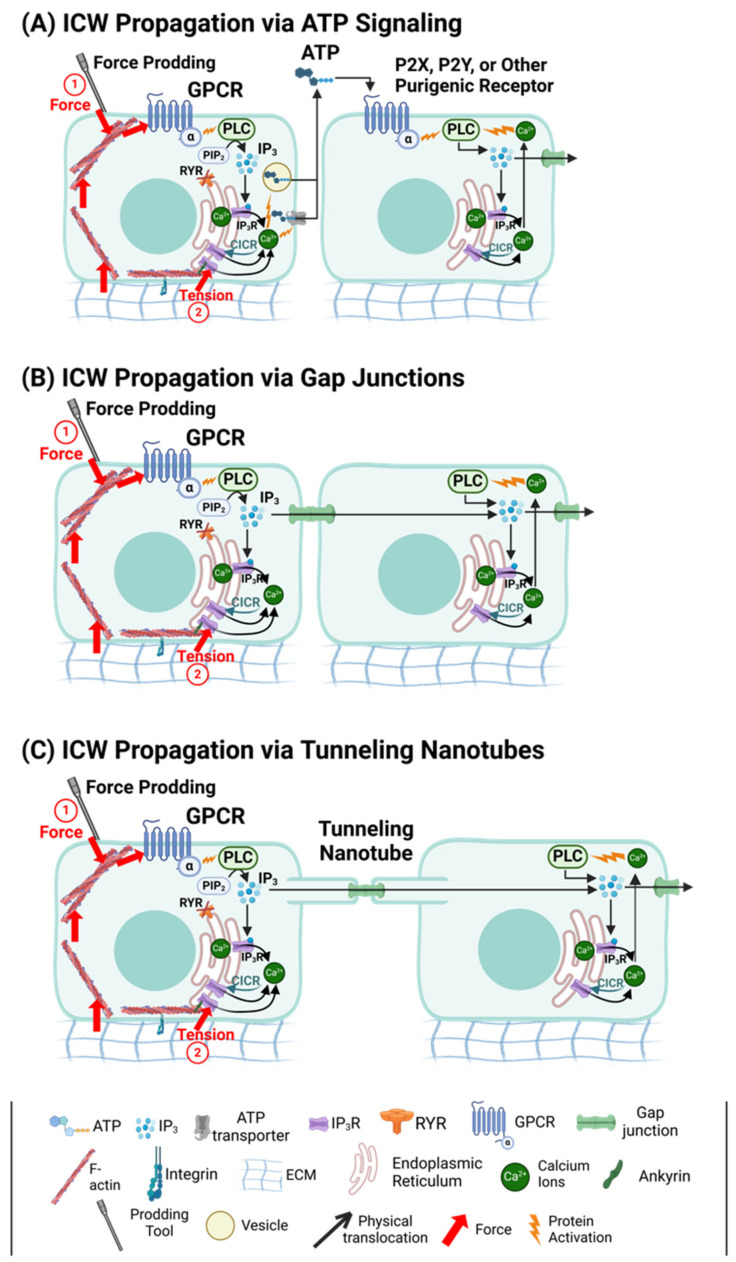
Mechanisms of ICW propagation. (**A**–**C**) Mechanical signals from force prodding and/or substrate tension activate GPCRs, triggering conformational changes in the α subunit which then activates PLC to cleave PIP_2_ into IP_3_ and DAG [46,60]. IP3 activates IP3Rs on the ER, stimulating the release of Ca^2+^ from the ER into the cytoplasm [45,137,138,147]. Cytoplasmic Ca^2+^ further sensitizes IP3Rs to IP3, inciting positive-feedback calcium-induced calcium release (CICR) [122,145]. Mechanical stimulation may also directly trigger ER Ca^2+^ release by transmitting force through cytoskeletal proteins like ankyrins, which link to IP_3_Rs or other mechanosensitive channels, inducing their activation [79]. (**A**) ICW propagation via ATP signaling. Increased cytoplasmic Ca^2+^ stimulates extracellular vesicles and ATP transporters to release ATP into extracellular space, where it acts as a long-distance signaling molecule by binding to purinergic receptors (P2X, P2Y) on receiving cells, causing subsequent Ca^2+^ release by stimulating the PLC-IP3-IP3R- Ca^2+^ pathway [53,74,109,144,155]. (**B**) ICW propagation via gap junctions. IP3 signaling molecules pass directly from an initiating cell to an adjacent receiving cell through gap junctions, stimulating intracellular Ca^2+^ release by binding to IP3Rs on the receiving cell’s ER. Rising levels of cytoplasmic Ca^2+^ activate the PLC-IP3-IP3R-Ca2 pathway, driving further intracellular Ca^2+^ release [83,85,143,144,156]. (**C**) ICW propagation via tunneling nanotubes. IP3 signaling molecules travel from initiating to receiving cells via tunneling nanotubes (TNTs) connected by gap junctions, initiating calcium release in the receiving cell via the PLC-IP3-IP3R-Ca2 pathway and CICR [146,147,148].

ICWs propagate through a combination of mechanisms that ensure efficient communications between cells and tissues [109]. After intracellular calcium is released into the cytoplasm, it can initiate ICWs via two primary methods: (1) ATP release into the extracellular environment (Figure 3A) or (2) direct transfer of signaling molecules through GJs (Figure 3B). In the ATP-mediated pathway, calcium stimulates the diffusive release of ATP, which binds to purinergic receptors (e.g., P2X, P2X2, P2Y, etc.) on neighboring cells, triggering calcium release in those cells. This method is typically faster (*v_avg_* = 7 μm/s) but is often limited to shorter distances (100 μm) [142]. Conversely, GJs allow for the direct transfer of IP3 molecules between adjacent cells, facilitating slower but longer-range signal propagation. As IP_3_ binds to IP_3_Rs in adjacent cells, it triggers ER Ca^2+^ release and CICR to propagate the wave [143,144]. The resulting cytoplasmic Ca^2+^ can further activate PLCs, leading to additional IP_3_ production and amplifying the intercellular Ca^2+^ signal [145]. Another unique form of GJ-mediated communication occurs through tunneling nanotubes (TNTs; Figure 3C)-thin, actin-rich membrane extensions connected by GJs which bridge distances between cells, enabling ICW propagation via direct transfer of IP3 and Ca^2+^ [146,147,148].

In addition to these classical transmission pathways, ICWs can also propagate further distances through regenerative wave spread that involves active signal amplification by downstream cells [109]. ICWs have also been shown to spread via less-investigated paracrine mechanisms involving molecules other than ATP, including nitric oxide [149,150] and prostaglandins [151]. In astrocytes, NO promotes the propagation of ICWs through distinct pathways depending on the types of stimulation. Micropipette poking can trigger NO production, which induces plasma membrane depolarization and activates L-type voltage-gated Ca^2+^ channels, enhancing Ca^2+^ influx and supporting ICWs spread [152]. Glutamate stimulation activates metabotropic glutamate receptors (mGluRs), leading to eNOS-derived NO that directly S-nitrosylates CALHM1 channels, resulting in ATP release and downstream purinergic receptor-dependent activation of Cx43 and Panx-1 channels, and amplifying and coordinating calcium transients with wave-like propagation [153].

The speed and propagation distance of ICWs depend on several interacting factors, including the initiation mechanism (mechanical or non-mechanical) [76,81], mode of spread (e.g., ATP release, gap junctions, tunneling nanotubes) [142,143,144], presence of regenerative wave amplification [109], cell type [53,89,90], and the spatial organization of the cell group (e.g., monolayer vs. 3D tissue) [53,154]. Because these properties arise from a wide variety of interacting biophysical factors, currently there is no precise way or formula known to accurately pre-determine ICW speed and distance. The exact pathway or formula remains to be investigated. The following Section 2.3.2, Section 2.3.3, Section 2.3.4, Section 2.3.5, Section 2.3.6 discuss each mechanism in detail.

#### 2.3.2. ATP-Mediated ICW Spread

Various studies have shown that ICWs’ long-range spatial propagation is critically dependent on the spatially diffusive release of ATP, known as ATP waves (Figure 3A). These ATP molecules function as endocrine signaling molecules to initiate and sustain ICWs [81,85,93,109,147,157,158,159]. P2 purinergic receptors P2X and P2Y are commonly shown to play a role in propagating ICWs by accepting extracellular ATP and inducing downstream Ca^2+^ activities [142].

For instance, the addition of ATP-degrading apyrase abolishes ICWs in human mesenchymal stem cells (HMSCs) [79], while both apyrase or purinergic receptor blockers Suramin and PPADS abolish ICWs in FUS-stimulated PC-3 human prostate cancer cells [88]. Purinergic receptor antagonists, ATP depletion, and ATP scavenging each completely inhibit ICW propagation in DU-145 prostate cancer cells, suggesting a wave propagation mechanism reliant on ATP release, mechanosensitive anion channels, and purinergic ATP receptors [76]. Stanniocalcin-1 (STC1) regulates ICWs in human pulmonary (A549) and prostate (PC3) epithelial cells by stimulating ATP release from bystander cells, indicating a cooperative mechanism for promoting ICW spread [74].

Extracellular ATP injection is another common method of triggering ICW initiation independent of mechanical stimulation, highlighting the pivotal biochemical roles that ATP can play in initiating ICWs. This approach highlights the sufficiency of ATP in generating ICWs, even in the absence of other potential contributing factors. For instance, exogenous ATP application has been shown to reliably initiate ICWs in various cell types, including astrocytes, endothelial cells, and epithelial cells [81,85,142]. These results not only underscore ATP’s central role in ICW initiation but also provide a reproducible means of studying calcium signaling dynamics across diverse cellular environments.

#### 2.3.3. GJ-Mediated ICW Spread

GJs are specialized intercellular channels composed primarily of connexin43 (Cx43; Figure 3B), which assembles into hexameric connexons that dock between adjacent cells to facilitate selective cytoplasmic exchange of calcium ions and small signaling molecules [85,86,156,160]. GJs facilitate the spread of the calcium wave, ensuring synchronous cellular responses throughout the tissue. In various studies, the inhibition or blocking of GJs with chemicals including octanol [142,157], carbenoxolone [122,142], heptanol [85], and halothane [82] result in decreased ICW spread or duration, indicating the dependence of ICW spread on GJs in these conditions.

Studies on HeLa M-sec cervical cancer cells [147], H-SY5Y neuroblastoma cells, and HEK cells [148] indicate that passive Ca^2+^ diffusion through GJs is insufficient for ICW propagation, with IP_3_ transfer serving instead as the primary mediator [122]. The IP_3_ signaling pathway amplifies intracellular waves by increasing IP_3_ levels in adjacent cells, triggering calcium release from the ER and sustaining signal propagation over longer distances [86,145,161]. Several recent studies demonstrate GJ-reliant ICW mechanisms. ICW propagation (*v* = 12.1 ms^−1^) in NIH-3T3 mouse fibroblast cells was abolished completely with octanol, a Cx43 inhibitor [157]. ROS 17/2.8 rat osteosarcoma cell lines, which express Cx43 and lack P2Y2 receptors, were shown to transmit slow (7.4 µm/s) GJ-dependent ICWs [144]. As calcium signals propagate through the tissue, ATP may be released from PANX1 hemichannels, acting as a secondary messenger [76,88]. ATP activates purinergic receptors (P2X, P2Y) on neighboring cells, further amplifying calcium release from their ER stores and perpetuating the calcium wave, thus ensuring the synchronization of collective cellular responses [158,162].

#### 2.3.4. Combined ATP- and GJ-Mediated Mechanism

Notably, these mechanisms are not mutually exclusive but can be simultaneously implemented by cells (Figure 3). Their relative contribution is dynamically modulated based on cell types, connexin expression, ATP availability, and microenvironment, enabling efficient communication between cells and tissues through the propagation of ICWs. ICWs in HeLa cells propagate via both ATP-mediated extracellular signaling and GJ-mediated intracellular pathways, with the dominant mechanism depending on connexin expression and ATP availability [45]. Bone cells largely rely on an ATP-diffusion-dependent mechanism for propagating ICWS, with GJs playing a significantly lesser role [27], whereas astrocytes preferentially utilize the GJ-IP_3_ pathway to mediate calcium wave propagation [117]. In the adult mouse organ of Corti, two separate types of ICWs were identified with distinct propagation mechanisms: slow (1–3 μm/s), periodic, GJ-mediated ICWs propagating longitudinally along the cochlea were inhibited by GJ blockers 1-octanol and carbenoxolone, while fast (v = 7 μm/s) ICWs stimulated by extracellular ATP were modulated by P2 receptor antagonists PPADS and suramin, likely mediated by an ATP-dependent mechanism involving activation of P2X2 purinergic receptors identified in supporting cells and outer hair cell stereocilia [142].

While most cell lines rely on ATP- and GJ-dependent mechanisms for ICW propagation, few exhibit ICW spread without either pathway, underscoring their essential roles in cellular communication. However, some smooth muscle cells may propagate calcium waves through a distinct ECM-stiffness-dependent mechanical signaling mechanism independent of gap junctions and extracellular diffusion, posing a unique exception to the rule [114].

#### 2.3.5. Tunneling Nanotubes

Tunneling nanotubes (TNTs) represent a specialized extension of GJ-mediated ICW propagation (Figure 3C). They maintain GJ connectivity while enabling signal transmission over semi-long distances (approx. 10–70 µm) [146,147,148,160]. These dynamic, actin-based membrane protrusions establish direct cytoplasmic continuity between distant cells, facilitating the transfer of ions and signaling molecules such as Ca^2+^ and IP_3_. TNTs also enable the transfer of larger cargoes, such as organelles, between cells, and can adapt to cellular stress, as seen in astrocytes [108,163].

Like in GJ-mediated ICW propagation, photo-released IP3 experiments show that IP3 diffusion through TNTs, rather than the direct transfer of Ca^2+^, is a key driver of intercellular Ca^2+^ signaling [147]. TNTs have been directly implicated in ICW propagation in multiple cell types, including HeLa M-Sec cells [147,164] and SH-SY5Y cultured neuroblastoma cells [148]. TNTs have been found in Wnt/Ca^2+^ communication between neurons [108], which is significant because a noncanonical, β-catenin-independent [165,166,167,168] Wnt-PLC-IP3-Connexin-Ca^2+^ signaling axis has been demonstrated to play an important role in influencing ICWs in zebrafish spinal cords [169] and cardiac myocytes [168]. Together, these findings highlight the multifaceted nature of TNT-mediated communication, bridging the gap between direct cell-to-cell connections like GJs and broader signaling mechanisms like ATP waves.

#### 2.3.6. Regenerative Wave Spread

Regular propagation mechanisms of ICWs mainly include ATP-, GJ-, and TNT-mediated modalities, and may include other pathways such as prostaglandins or NO. The initiation mechanisms of these propagations typically rely on exogenous signals, such as mechanical stimuli, chemical stimuli, or regulatory factors from neighboring cells (e.g., STC1). Upon activation by an initial mechanical stimulus, such as mechanical poking or mechanical microenvironmental influences, paracrine biochemical signals (such as ATP or NO) are transmitted to adjacent cells via one or more of these pathways, facilitating intercellular communication. Unlike the unidirectional transmission of propagation, the regenerative waves are amplified by the downstream cells that autonomously boost and relay the signals. The speed and duration of the regenerative waves depend on the tissue structure and cell type, and it possesses greater robustness and adaptability over longer distances (up to thousands of cells). A recent report suggests that the influences of mechanical microenvironments, not mechanical poking, can regulate the magnitudes of wave speeds and wave distance, indicating previously understudied roles of mechanical inputs in mediating regenerative and trigger waves [53]. This phenomenon is supported by experimental and modeling studies that reveal its underlying mechanisms and implications for efficient and robust signal transmission across cellular networks [87,109,122].

MacDonald et al. (2008) developed a diffusion model demonstrating that regenerative ATP release from downstream astrocytes significantly enhances ICW propagation. The dual signaling model—combining initial point-source ATP release and downstream regenerative release—provides an efficient and energetically robust mechanism for intercellular communication, aligning well with observed spatiotemporal kinetics [109]. In *Drosophila* imaginal discs, wavefronts of ICW maintained their strength over several thousand cells, suggesting cell-autonomous regeneration of the ICW signal via a GJ-mediated mechanism [122]. Similarly, intermediate cells in micropatterned BV-2 microglial assemblies can act as regenerative amplifiers, enhancing ICW transmission to outermost cells [87]. Together, these findings highlight regenerative wave propagation as a fundamental mechanism for sustaining and enhancing long-distance ICW transmission, ensuring efficient and robust signal spread across diverse cellular systems.

## 3. Physiological Roles of Calcium Waves and ATP Signals

### 3.1. Cancer Cells

Ca^2+^ signaling plays a well-established role in physiological processes essential for tumor progression [2,170,171,172,173]. As a specialized form of Ca^2+^ signaling, ICWs enable coordinated cellular responses with tumorigenic roles [115], including metastasis [161], migration and invasion [174], and tumor growth [53].

In many cases, ICWs have been proven as drivers of tumorigenesis and related pathological processes. For instance, in human HCT-8 colon cancer cells, mice inoculated with IP3-sponge-transduced cells—which showed a 16-fold reduction in spontaneous calcium transients and ICWs compared to controls—developed tumors with 1.6 times lower weight, demonstrating a causal role of ICWs in driving tumorigenesis in vivo [53]. In oncogenic RasV12-transformed mammalian epithelial cells and zebrafish embryos, ICWs initiated by a transformed cell induced apical extrusion and polarized movement of surrounding cells through an IP3R-GJ-TRPC1-mediated mechanism [27].

In other cases, ICWs act as biomarkers for cancer, though an unexplored causal role may still be at play. Scratch-wound assays in wild-type C1 CRC cells induce extracellular Ca^2+^ influx through voltage-gated calcium channels (VGCCs), driving initial calcium transients and promoting BCL9 interaction with paraspeckle proteins. Subsequently, BCL9 translocation into paraspeckles promotes sustained ICW propagation, cytoplasmic projections, tumor progression, tissue remodeling, and stromal cell infiltration [175]. Studies using FUS have revealed that invasive cancer cell lines (e.g., PC-3, MDA-MB-231) exhibit robust calcium oscillations in response to stimulation, whereas non-invasive cells of the same type do not. This suggests that invasive cells may possess intrinsic properties that make them particularly receptive to calcium signaling, potentially enhancing their metastatic capabilities [88,176]. The underlying mechanisms remain to be investigated.

Despite growing evidence linking ICWs to the promotion of tumorigenesis, some studies suggest modulated calcium signaling—including calcium waves—may suppress tumors via induction of apoptosis [177,178,179]. Electromagnetic fields modeled after ICWs have been shown to induce apoptosis in B16-BL6 melanoma cells, possibly indicating a tumor-suppressive role of ICWs in some cell lines [146]. Given that ICWs often propagate through extracellular ATP signaling, studies implicating ATP waves in cancerous phenotypes may, in part, reflect underlying ICW activity that has yet to be fully characterized [155]. Further investigation into the mechanisms and context-dependent functional effects of ICWs could provide critical insights into their potential as new therapeutic targets or biomarkers in oncology.

### 3.2. ICWs in Non-Cancer Cells

In non-cancerous biological systems, ICWs mediate various critical functions [42,44] including neuronal plasticity [113,117,118], stem cell differentiation [123,180], organ developmental regulation [92,94], tissue repair [120,181,182], viral priming [183,184], and muscle contraction [80,84,115,116].

In neurons, ICWs have been shown to regulate neuronal division in the ventricular zone of the neocortex [113]. Substrates’ mechanical rigidity regulates Ca^2+^ oscillation via the RhoA pathway in HMCSC stem cells, potentially influencing cell differentiation [123]. In *Drosophila* wing imaginal discs, ICWs exhibit spatiotemporal patterns that regulate organ growth through calcium spikes, transients, and waves. Perturbations to these signals can result in abnormal organ sizes, with either excessive or insufficient calcium signaling reducing growth [92,94]. ICWs mediate wound healing, as seen in polarized hepatic cells where ICWs guide preferential cell growth toward the wound edge [181]. In epithelial tissues, regenerative calcium waves traverse thousands of cells, enabling synchronized responses such as cell extrusion [27] and tissue repair [182]. Similarly, mechanical injury in *Drosophila* wing discs triggers slow IP3R-mediated ICWs, promoting tissue repair [120]. Calcium is also shown to regulate patterning and actomyosin organization in the *Drosophila* disc epithelium [122]. Rotavirus infection induces ICWs through ADP signaling, effectively priming surrounding cells for rapid viral spread [183,184]. In smooth muscle cells, the influx of calcium ions activates myosin light chain kinase (MLCK) to trigger contraction, while modulation of ATP-sensitive potassium channels regulates membrane potential and excitability [84]. These coordinated propagations of calcium and ATP signals are essential in urothelial cells, helping regulate their responses to mechanical and metabolic stress [85].

The interactions between mechanosensitive molecules and calcium signals underpin a myriad of pathological processes and therapeutic treatments. For example, malocclusion is a highly prevalent dental disease and severely impacts on oral health, such as causing periodontitis. Periodontitis increases the risk of general systemic diseases, including respiratory tract infections, Alzheimer’s disease, diabetes, and cardiovascular diseases [185,186]. Mechanotransduction via calcium influx and mechanoreceptor-induced signaling are critical steps during orthodontic tooth movement (OTM) for treating patients with malocclusions [187]. Mechanoreceptors located on the cell membrane, such as ion channels Piezo1, can receive mechanical forces and convert the signals to activate intracellular signaling, such as yes-associated protein (YAP) [188]. Researchers show that Piezo1 can increase intracellular calcium levels, leading to the dephosphorylation and nuclear translocation of YAP, transforming it into a transcriptional co-activator in the nucleus under mechanical stimulus [189,190]. Furthermore, Piezo1 controls the YAP-dependent expression of type II and IX collagens in osteoblasts under mechanical loads and influences osteoclast development [191,192], while YAP could regulate Piezo1 expression in turn. Nuclear localization of YAP could activate Piezo1 and enhance osteogenesis, and YAP, in collaboration with the G-protein-coupled estrogen receptor pathway, suppresses Piezo1 activation [193]. Some researchers show that Piezo1 exerts a signaling transduction role in mechanical stress-induced osteoclastogenesis, in which the markers cyclooxygenase-2, receptor activator of NF-kB ligand, and prostaglandin E2 were significantly upregulated under 2 g/cm^2^ static compressive loading for 0.5, 3, 6, and 12 h [194]. Piezo1 can also regulate periodontal ligament stem cell (PDLSCs) differentiation during OTM. Moreover, heavy mechanical forces up-regulate Piezo1 in PDL cells, reducing mitochondrial calcium influx and leading to the reduced cytoplasmic release of mitochondrial DNA. The subsequent signals inhibit the activation of the cGAS-STING signaling cascade and monocyte-to-osteoclast differentiation. Suppression of Piezo1 or up-regulation of STING expression under heavy mechanical force significantly increases osteoclast activity and accelerates OTM [195]. Furthermore, Piezo1 deficiency in osteoblastic cells leads to loss of bone mass and spontaneous fractures with increased bone resorption [190]. Piezo1-deficient mice are resistant to bone loss and bone resorption induced by hind limb unloading, demonstrating that Piezo1 can affect osteoblast–osteoclast crosstalk in response to mechanical forces [191].

### 3.3. Physiological Conclusions and Therapeutic Targets

Ultimately, ICWs play a fundamental role in mediating diverse physiological and pathological processes, from organ development and tissue repair to viral priming and muscle contraction. Their mechanosensitive nature enables precise regulation of multiscale cellular responses, particularly through calcium signaling pathways.

The intricate interplay between mechanical forces, calcium influx, and intracellular signaling cascades highlights the potential for targeted therapeutic interventions leveraging the mechanosensitive properties of ICWs and their physiological effects. Relevant mechanobiological strategies for cancer treatment include targeting proteins necessary for ECM stiffness (e.g., TGF-β, collagen), ECM crosslinking (e.g., Pan LOX, LOX, LOXL2), ECM mechanosensors and mechanotransducers (e.g., integrins, Piezo channels), and nuclear mechanotransduction through interventions including drug treatment, mechanical stretch, and low-intensity pulsed ultrasound (LIPU) [196,197,198].

Targeted manipulation of key molecular components, such as Piezo1, a mechanosensitive ion channel that controls ICW dynamics, provides an avenue for controlling ICW dynamics and selectively inducing antitumor responses. In prostate cancer cells, compression of the cell membrane by a glass probe activates Piezo1, which enhances ICW and subsequently activates the Akt/mTOR signaling cascade, promoting cancer cell proliferation, migration, and cell cycle progression. Knockdown of Piezo1 by shRNA or the Piezo1-specific antagonist GsMTx4 inhibited tumor growth in vitro and in vivo in DU145 cells and xenograft nude mouse models [199]. Similarly, cyclic mechanical stretch induces ICWs in Tpm2.1 knockdown cells via Piezo1, leading to calpain–2-mediated apoptosis. Restoration of the actin-regulatory protein Tpm2.1 suppressed ICWs and reversed the apoptotic response [200].

In addition to molecular targets, mechanical stimuli, including bubble-jetting flow and FUS, have been shown to be effective in modulating ICWs, providing a potential platform for non-invasive mechanotherapy of tumors. These stimuli may modulate calcium-sensitive apoptotic pathways and alter gene expression, thereby altering cancer cell viability, invasiveness, and response to therapies. Using laser-induced tandem bubble-jetting flow to stimulate single HeLa cells induces integrin-mediated ICWs that modulate intracellular calcium signaling pathways linked to cytoskeletal remodeling and membrane repair, inspiring a targeted mechanotherapeutic strategy to regulate cells using ICW without compromising membrane integrity [89]. In murine B16F10 melanoma cells expressing the mechanosensitive channel of large conductance (MSCL), localized ultrasound mechanical stimuli trigger the activation of MSCL, leading to sustained calcium influx that induces apoptosis via ER stress and a downstream calpain–caspase–3 pathway. Those methano-regulated apoptotic tumor cells released antigenic debris that promoted dendritic cell (DC) maturation and CD8^+^ T cell activation, resulting in a robust and tumor-specific immune response [201].

## 4. Cutting-Edge Technologies for Ca^2+^ and ATP Imaging

### 4.1. Functional Imaging of Ca^2+^ Dynamics

**Figure 4:**
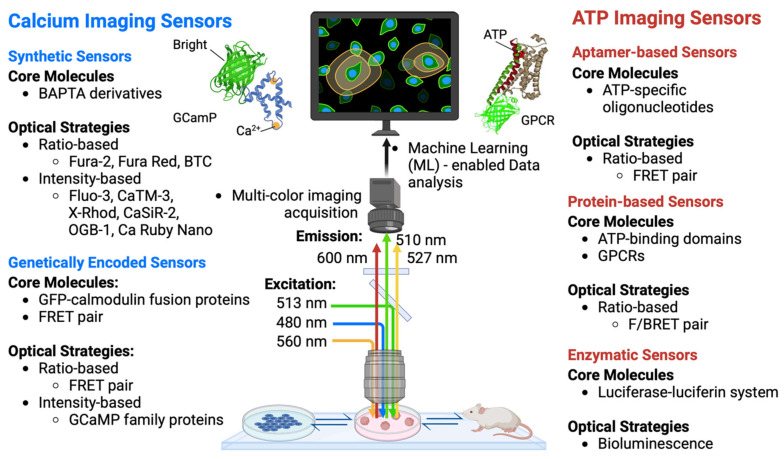
The sensors for optical imaging of calcium and ATP dynamics. A schematic representation of the core components required for imaging calcium and ATP signals is shown. Calcium imaging utilizes both synthetic and genetically encoded sensors, with optical strategies including ratio-based and intensity-based approaches. ATP imaging involves aptamer-, protein-, and enzymatic-based sensors with ratio-based (F/BRET) and bioluminescence optical strategies. Different sensors use specific excitation and emission wavelength lasers, enabling multi-color imaging acquisition. The upper left diagram illustrates a representative class of calcium sensors, the GCaMP family proteins. The upper right diagram represents an ATP sensor, GPCRs, adapted from Lin, L, licensed under CC BY 4.0 [209]. Machine learning-enabled data analysis further refines the detection and interpretation of signals, providing precise dynamic imaging.

Given the importance of calcium ions in various physiological processes, there have been continuous research efforts in the last few decades to develop and improve fluorescence sensors for optical calcium imaging (Figure 4) [53,202,203,204,205]. Synthetic calcium sensors used today are mostly derivatives of 1,2-bis(o-aminophenoxy)ethane-N,N,N′,N′-tetraacetic acid, or BAPTA [206,207,208]. Compared to an ethylene glycol tetraacetic acid (EGTA) moiety, BAPTA has the advantage of an optical readout change upon calcium binding, fast binding kinetics, and independence from pH, while maintaining its high selectivity for calcium ions. Binding/chelating of calcium to BAPTA results in a shift from an “aniline-like” structure to a “benzene-like” structure that can be exploited for fluorescence property changes.

Broadly speaking, there are two parallel strategies in modulating optical readout changes for calcium imaging (Figure 4): (1) ratio-based indicators and (2) intensity-based indicators. Ratio-based indicators change the shape of their absorption or emission spectra. Upon binding between calcium ions and ratio-based indicators, the ratios of peak fluorescent intensity between two wavelengths change (e.g., fura-2, Fura Red, and BTC) [210,211,212]. In contrast, intensity-based indicators retain the overall shape of their absorption and emission spectra, but their fluorescence intensity typically increases upon binding between calcium ions and indicators (e.g., fluo-3, OGB-1, CaTM-3, Ca Ruby Nano, X-Rhod, CaSiR-2) [213,214,215]. While both strategies are widely implemented today, intensity-based indicators are relatively more appealing due to their ease of use, as they circumvent complex microscopic illumination, detection schemes, and ratiometric calibration.

Another class of calcium sensors gaining desired traction in cell biology and animal research is genetically encoded calcium indicators (GECIs) [216,217,218]. The GECIs are engineered proteins that exhibit a change in fluorescence upon physical binding between proteins and calcium ions. Because GECIs can be stably expressed in cell lines and transgenic animals, they have the advantages in biocompatibility and ease of use by eliminating the need to stain and wash, unlike their synthetic counterparts. GECIs are read out based on changes in fluorescent color ratio or intensity. To modulate changes in color ratio, a protein engineering strategy is to develop Förster resonant energy transfer (FRET) sensors where a FRET pair of fluorescence proteins is combined with a calcium binding module that changes conformation upon calcium binding to either facilitate or inhibit FRET [219,220]. Regarding the changes in intensity, a representative class of calcium sensor is the GCaMP family proteins. They are a fusion protein of circularly permuted green fluorescent protein (cpGFP) and Ca^2+^-binding calmodulin. When calcium ions bind to calmodulin, calmodulin changes the local microenvironment of the fluorophore in neighboring cpGFP and increases its fluorescence [221].

As the utility of calcium indicators is expanded through simultaneous imaging of multiple different proteins of interests, recent research shows rapid progress in expanding the color palette of calcium fluorescence indicators. While most of the early generations of synthetic and genetic calcium indicators emit green fluorescence, red/yellow-emitting indicators such as those in the RCaMP family have been developed to offer multiplexed imaging capabilities [222,223]. These advances hold the potential to image diverse ICWs in multiple distinct tissues and cell types simultaneously, leading to the decoding of multiscale intercellular calcium language in living systems.

Another important consideration in choosing the appropriate calcium sensors for imaging is the response time of the sensors compared to the ICW propagation speed. Recent advances in GECIs have seen accelerated response time on the order of 50–100 milliseconds, approaching to capturing a single electrical action potential [224,225]. Given that the fastest ICW speed is ~10–30 µm/s [53], which is equivalent to about one cell per second during propagation, those faster GECIs are well equipped to capture the ICW dynamics, such as fast GCaMP3 [226], GCaMP6f_u_ [227], and GCaMP6f [228]. For high-resolution fluorescence imaging of ICW, the spatial resolution per the diffraction limit is on the order of ~300 nm [229]. The mammalian cell size is one or two orders of magnitude larger than the diffraction limit, making high-resolution fluorescence imaging a facile and sufficient platform for monitoring ICW propagation across cells.

### 4.2. Functional Imaging of ATP Dynamics

Extracellular ATP sensors are molecular tools designed for accurately measuring concentrations of ATP molecules across various biological contexts. These tools provide the capabilities to monitor real-time secretion and diffusion/reaction dynamics of ATP, leading to new insights into intracellular signaling and pathological processes. These biosensors rely on specific biorecognition elements and signal transduction mechanisms. The most notable two types are (1) binding-based and (2) enzymatic-reaction-based sensors, each employing distinct strategies for ATP detection.

**Figure 5:**
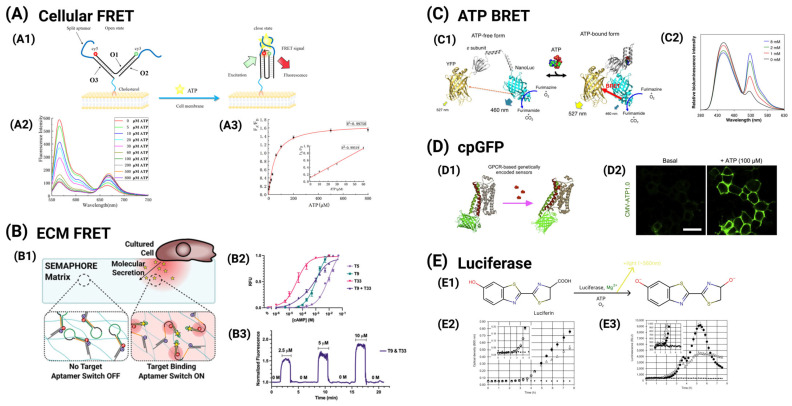
(**A**) Depiction of the mechanism behind the ratiometric DNA nanoswitch anchored to the cell surface used for imaging extracellular ATP (**A1**) [230]. The fluorescence spectra illustrate the response of the DNA nanoswitch to various ATP concentrations in vitro at 37 °C, showcasing the FRET ratio of FA/FD in relation to ATP concentrations (**A2**). The inset presents the calibration curve for concentrations ranging from 5 to 60 μM (**A3**). (**B**) Cells are cultured directly on the SEMAPHORE matrix [234]. Secreted signaling molecules diffuse into the hydrogel, where they engage with target-specific aptamer switches, resulting in localized fluorescent signaling. The fluorescence response of the SEMAPHORE system, which contains cAMP-responsive aptamer switches of varying poly-T linker lengths, is measured over time (**B1**). The plot illustrate the fluorescence response of the SEMAPHORE system (**B2**) and the time-resolved measurement of the SEMAPHORE fluorescent response (**B3**). The system exhibits rapid and reversible cAMP detection in response to varying concentrations of cAMP. (**C**) Schematic representation of BTeam [236]. In the absence of ATP, the ε subunit remains extended and flexible, which separates YFP and NLuc, leading to low BRET efficiency (**C1**). Conversely, the presence of ATP induces a conformational change in the ε subunit, bringing YFP and NLuc closer together, thereby enhancing BRET efficiency (**C2**). It is important to note that the ε subunit can reversibly bind and release ATP without hydrolysis. The ATP-dependent luminescence spectral changes of purified BTeam are also illustrated. (**D**) Illustration of the principles behind GRAB-based ATP sensors, which utilize the human P2Y1 receptor as a scaffold linked to circularly permuted enhanced green fluorescence protein (cpGFP). ATP binding initiates a conformational change that enhances the fluorescence signal. (**D1**) The illustration of sensor composed of a conformationally sensitive circularly permutated GFP (cpGFP) and a ligand-binding protein [209]. (**D2**) Representative fluorescence images of HEK293T cells expressing the ATP1.0 sensor are presented, demonstrating both basal conditions and the response to the presence of 100 μM ATP [237]. (**E**) Overview of ATP measurement using luciferase assays [243]. Bacterial growth in liquid media is monitored through optical density assessments (**E2**) and luminescence measurements (eATP, utilizing thermostable luciferase and D-luciferin) (**E3**). Filled circles represent S. aureus, open triangles denote *E. coli*, and filled diamonds indicate the sterile control. The initial cell density was 100,000 CFU/mL.

Aptamer-based biosensors utilize ATP-specific oligonucleotides as recognition elements [230,231,232,233]. They typically incorporate FRET motif to translate binding events into quantifiable signals (Figure 5A,B). When ATP binds to the aptamer, it induces oligonucleotide structure-switching that alters the proximity of a fluorescent dye to a quencher or enhances interactions between FRET pairs [234,235], resulting in a measurable fluorescence intensity shift that directly correlates with ATP concentration. Aptamer-based sensors are versatile, suitable for real-time monitoring on various substrates, including cell surfaces and hydrogels, and exhibit high specificity and speed (Figure 5C). Alternatively, recombinant-protein-scaffolds-containing-based sensors use engineered proteins, such as ATP-binding domains [236] (Figure 5D) or GPCRs [237] (Figure 5E), to detect ATP. These sensors employ either fluorescence/bioluminescence resonance energy transfer (F/BRET) [236] (Figure 5D) or circularly permuted FP (similar to the design strategy of GCaMP sensors) [238] as their signal transduction mechanisms. When ATP binds to the recognition site, it triggers a conformational change in the protein, which either affects the configuration of fluorescent proteins [239,240,241] tagged to the ATP binder or alters the distance between bioluminescent/fluorescent dyes and their FRET partners [242]. This change produces a detectable optical signal that can be correlated with ATP concentration.

Another prominent method for ATP detection is the enzymatic reaction-based approach [243,244,245], notably those that employ the luciferin/luciferase system [243]. These biosensors exploit biochemical reactions where the luciferase catalyzes the conversion of luciferin in the presence of ATP, resulting in light emission that serves as a quantifiable measure of ATP concentration.

The luciferase enzyme binds to both luciferin and ATP, facilitating a reaction that generates an oxidized form of luciferin that emits light [244]. This bioluminescent signal is highly sensitive and characterized by low background interference, making it an effective method for detecting ATP. However, despite its sensitivity, this method does have limitations, including their restricted dynamic range and dependence on the availability of luciferin, which can affect performance.

### 4.3. Artificial Intelligence (AI)/Machine Learning (ML)-Enabled Data Analysis

To effectively interpret the recorded spatial-temporal dynamics of cellular signals including both calcium/ATP and electrical spikes, computational techniques such as denoising, signal decomposition, and peak detection are often required to sequentially extract the information encoded in all firing dynamics (Figure 4). For many decades, researchers have been pursuing computational methods that can automatically detect spikes, including methods such as template matching [246,247]. The task of calcium imaging analysis is to identify calcium spikes from calcium imaging videos, which can be treated as a time series of brightness signals for each pixel in each frame. Unfortunately, this task is still challenging for many AI/ML models due to the complex inherent characteristics of the imaging data, such as non-linearity, heterogenous variations of cell size at pixel-level, decay of measured signals due to experimental limitations such as electrode drift or photo-bleaching, low signal-to-noise ratio (SNR), and the short duration of spiking events (which makes them resemble random noise). To the best of our knowledge, no existing method can universally and automatically identify all types of spikes. Therefore, we have been designing a case-by-case-based AI/ML pipeline to process and analyze neuronal spikes. However, modern AI/ML models typically require a large amount of human-labeled data to achieve good performance, demanding high levels of human labor and resources.

The typical design of an AI/ML pipeline in the field is as follows. First, imaging data are preprocessed by tailored signal processing techniques such as wavelet transform [248,249]. Second, machine learning methods are used to further process the data. For example, the principal component analysis (PCA) [250,251] enables identifying the projection direction of most variance. Third, all pixels that represent the objects of interest are clustered by methods such as K-means [252,253], the Gaussian mixture model (GMM) via expectation-maximization [254,255], or entirely manually labeled. With broader definition on spike signal detection, people use convolutional neural networks (CNN), recurrent neural networks (RNN), and long short-term memory (LSTM) [256] to find the spiking activities [257,258]. Although these newly developed deep neural networks are powerful, they have certain limitations. For example, their black-box nature makes it difficult to interpret the working mechanism, challenging to tune the model parameters, and computationally expensive to accomplish training and inference. Recent studies have applied this pipeline to ICW-related calcium imaging tasks. By combining calcium image preprocessing with spatial segmentation using fully convolutional networks, followed by temporal spike detection using long short-term memory (LSTM) models, calcium transients induced by mechanical stimulation can be accurately identified [259]. Additionally, 3D U-Net architectures can be directly applied to full-frame confocal imaging data to detect and classify local calcium release events automatically [260].

One of the fundamental challenges in applying AI/ML techniques of detecting ICW is the difficulty of obtaining large, high-quality labeled datasets. On the one hand, high-quality labeled spiking samples in calcium imaging are inherently expensive and difficult to obtain; detecting calcium spikes requires not only spatial segmentation but also precise temporal annotation of transient signal fluctuations embedded in noisy backgrounds. This process is highly labor-intensive and demands substantial domain expertise, and even trained experts may struggle to distinguish true spikes from noise. As a result, annotated datasets are typically small, costly to produce, and prone to inconsistencies due to subjective interpretations.

On the other hand, deep learning models are fundamentally data-hungry for the context of detecting ICW. To contextualize the data demands of AI/ML models, it is useful to revisit early deep learning systems. For instance, AlexNet, a seminal CNN architecture, contained approximately 60 million parameters and was trained on 1.2 million labeled images from the ImageNet dataset. In contrast, ICW calcium imaging datasets often contain only a few thousand manually labeled spiking events. Note that the parameter size of modern DL models is often much larger, resulting in a severe data bottleneck. Compounding the problem, annotation in biological imaging remains subjective and labor-intensive, limiting scalability and reproducibility. Moreover, precise detection of ICW spike is inherently more challenging than traditional image classification tasks like ImageNet. Models such as AlexNet were trained to classify well-defined, static images into different categories, with high inter-class variability and abundant clean labels. In contrast, ICW analysis must detect sparse, transient, low-SNR (signal-to-noise ratio) events at the pixel level across time, often with ambiguous ground truth and significant biological variability. Even with similar data volumes, these differences make the modeling task fundamentally harder, further amplifying the limitations of current deep learning methods.

Unlike the classical ML models, the limited interpretability of deep learning models also plays a critical role in this context. Consider a simple linear classifier of the form Wx+b, where each parameter has a clear and intuitive meaning: for example, the weight vector W defines the slope and orientation of the decision boundary, while the bias term b shifts the boundary across the input space. Of course, such linear models are far too simple to capture the nonlinear dynamics of calcium spikes in ICW data. However, they serve as a clear example of what true interpretability looks like: each model parameter directly corresponds to a specific feature’s influence on the decision, and the entire decision boundary can be understood in terms of slope and offset. In contrast, most parameters in a deep neural network, often numbering in the millions, lack such intuitive meaning. Their role in the final prediction is entangled, and largely opaque, making it difficult to trace how a particular input leads to a specific output. This opacity limits their scientific interpretability, particularly when the goal is to understand underlying biological mechanisms or design interpretable experiments.

Therefore, despite the promise of deep learning in modeling complex signal dynamics, its application to ICW research must be approached with caution and deeper consideration. Hybrid strategies, combining classical interpretable models (e.g., dictionary learning) with deep networks, or leveraging unsupervised/weak-supervised methods, may provide more pragmatic and sustainable paths forward under real-world data constraints.

Hence, we highlight a new method that combines the wisdom of both classical ML methods and deep learning methods. Classical ML techniques refer to conventional ML models like PCA or dictionary learning, which can uncover hidden patterns in data using interpretable mathematical principles with affordable computational cost. These techniques contrast with deep learning approaches, which, although powerful, often function as black boxes that are computationally intensive and difficult to interpret and train. Our method sequentially combines classical ML techniques and deep learning techniques. Following standard filtering and other signal processing methods, the novelty of our method is to use periodic dictionary learning [261] to find the periodic signal like a pre-selection, which is more computationally friendly thanks to the highly parallel nature of dictionary representation inference (one needs only matrix multiplication to inference the representation) [262]. We propose to train both a classical machine learning model and a deep neural network to recognize patterns of interest. One may use either one approach or combine both, depending on the final performance.

## 5. Summary and Outlook

As a fundamental mechanism that regulates cellular communication [55], neuronal survival [263], synaptic plasticity, and memory formation [264,265], ICWs have been extensively studied in electrically excitable neurons. This review synthesizes recent advances in understanding how electrically non-excitable cells transduce mechanical stimuli into ICW dynamics across molecular, cellular, and tissue scales. We focus our quantitative discussions on how multiple classes of mechanical forces, including those externally applied (e.g., ultrasound, pipette poking, or mechanical stretching) or intrinsically generated (e.g., matrix stiffness, cytoskeletal stress, and membrane tension), regulate three key stages of ICWs: initiation, propagation, and regeneration or relay. Mechanosensitive ion channels, such as Piezo1 and TRPM7, along with ER-localized PANX1 channels, transduce mechanical forces into calcium release, often via IP3-dependent ER store depletion. In the downstream process, ICWs propagate through ATP purinergic signaling or GJ-mediated IP3 diffusion. These signal pathways consequently drive metastasis, proliferation, and stemness in cancer cells, while regulating tissue repair, developmental patterning, and organ growth in non-cancer cells. To advance the study of ICW dynamics, we summarize the new technologies including genetically encoded calcium and ATP biosensors and AI/ML algorithms that enable real-time visualization and analysis of 2D/3D ICW propagation, linking it to mechanotransduction, gene expression (YAP/TAZ activation), and pathological processes.

In addition to summarizing the molecular mechanisms of ICW initiation and propagation, we review the major knowledge gaps that remain to be filled. First, the quantitative properties of ICWs, such as speed, duration, and spatial extent in response to different mechanical stimuli, require further investigations. Second, the role of IP3 signaling in mechanically triggered ICWs, particularly in how cytoskeletal tension affects IP3 production and calcium release, also needs to be further understood. Third, the feedback mechanisms between ICWs and gene expression remain unclear, including the factors that influence ICW properties and the downstream mechanisms by which ICWs further regulate the activity of transcription factors. Furthermore, the differences in ICWs’ behavior between cancerous and non-cancerous tissues need to be elucidated. This difference provides a key opportunity to develop a new framework to deepen understanding of the roles that ICWs play in disease progression and to identify novel therapies. Overall, addressing these questions will enhance our understanding of ICW mechanobiology and drive the development of novel clinical approaches for cancer and other mechanobiology-related diseases.
